# Exploration of the external and internal factors that affected learning effectiveness for the students: a questionnaire survey

**DOI:** 10.1186/s12909-023-04035-4

**Published:** 2023-01-23

**Authors:** Ding-Ping Chen, Su-Wei Chang, Annette Burgess, Brian Tang, Kuo-Chien Tsao, Chia-Rui Shen, Pi-Yueh Chang

**Affiliations:** 1 Department of Laboratory Medicine, Chang Gung Memorial Hospital at Linkou, Taoyuan, Taiwan; 2 Graduate Institute of Biomedical Sciences, College of Medicine, Chang Gung University, Taoyuan, Taiwan; 3 Medical Biotechnology and Laboratory Science, Chang Gung University, Taoyuan, Taiwan; 4 Clinical Informatics and Medical Statistics Research Center, College of Medicine, Chang Gung University, Taoyuan, Taiwan; 5 Division of Allergy, Asthma, and Rheumatology, Department of Pediatrics, Chang Gung Memorial Hospital, Taoyuan, Taiwan; 6 Faculty of Medicine and Health, University of Sydney School of Medicine, Education Office, University of Sydney, Sydney, Australia; 7 Faculty of Medicine and Health, University of Sydney School of Medicine, Sydney Health Education Research Network, University of Sydney, Sydney, Australia

**Keywords:** Learning effectiveness, Medical technology, Questionnaire, Willingness, Motivation, Extracurricular studies

## Abstract

Learning effectiveness may be affected by internal and external factors, including personal attitude, motivations, learning skills, learning environment and peer pressure. This study sought to explore potential factors on students who majored in medical technology. The 106 students who completed their internship at Chang Gung Memorial Hospital were enrolled in this study. A written questionnaire was analyzed to explore the relationship between potential factors and learning effectiveness. The strength of relationship between the outcome and each factor was evaluated using Spearman correlation coefficients. A multiple linear regression model was constructed to assess how those factors affected learning effectiveness altogether. The results indicated that the learning effectiveness of the students mainly depended on three factors: the “extracurricular studies” and “willingness to cooperate” were positively associated with learning effectiveness. However, the “weakened motivation due to uncertainty” is negatively associated with learning effectiveness. We suggested that the educators can understand the uncertainty of students about the future. Additionally, the projects that require joint cooperation and discussion need to be given. The most important thing is that students should be able to integrate the learning content instead of rote.

**Supplementary Information:**

The online version contains supplementary material available at 10.1186/s12909-023-04035-4.

## Introduction

In recent years, annual average less than 20% of the college graduates in Taiwan have passed the medical technologist licensure exam [1]. In addition, workload of medical technologists is commonly disproportionate to their income. The harsh employment condition may reduce a novice’s motivation, increase pressure, and thus affect their learning effectiveness. Nowadays, there are many educational methods available to train medical technologist interns. However, how and what to evaluate the effectiveness of training of medical technologists remains to be elucidated. It is worth investigating that what factors may influence a student’s learning motivation, and what factors directly or indirectly affect learning effectiveness.

Motivation, learning approaches, stress, burnout, and some personal characteristics may affect learning effectiveness. Motivation plays an important role in learning effectiveness. Learning motivation, defined as an internal drive that activates behavior and gives it direction, occupies a very important position in the entire learning process [2]. Kasworm and Marienau considered “learning motivation” supports the learning goals [3]. In recent, the research result of Zhang and Chen [4] also indicated that learning motivation was the most critical force driving to learn, which helps learners actively participate in learning content. In addition, the research indicated that students’ learning motivation may also interact with the learning approaches they adopted [5]. Learning approaches is derived into deep, surface, and strategic approach, and which one approach adopted by student will be associated with their learning effectiveness [6]. And the learning approaches may change according to the examination mode, teachers’ teaching methods, peer influence or personal characteristics, such as attitude, and then affect the learning effectiveness [7, 8]. Stress may reduce academic performance and cognitive performance [9]. Although moderate stress could promote memory formation, excessive stress would have a negative impact on memory recovery and learning [10]. Additionally, stress does not affect all students equally [11]. Thus, personal characteristics are also a necessary item to conclude. Additionally, the excessive and unremitting stress may induce a state of emotional, physical and mental exhaustion, which is known as burnout [12]. Therefore, the Strength of Motivation for Medical School (SMMS) was used to measure the motivation of students [13], the Approaches to Learning and Studying Inventory (ALSI) was used to understand how a student approaches an academic task and predict the learning effectiveness by their learning approaches [14], the Perceived Medical School Stress (PMSS) was used to measure the stress level of students [15], and the Maslach Burnout Inventory-Student Survey (MBI-SS) is a scale used to evaluate the burnout for various countries and various professions students [16]. According to the above literature, it was demonstrated that these variables were important for learning effectiveness and had the potential to influence each other.

In order to improve the low passing rate of licenses in medical technology students, we wanted to understand what affects the learning effectiveness. And we assumed that any external and internal factors may affect learning effectiveness. Thus, the aim of this study was to explore potential factors that had an influence on the learning effectiveness of students who majored in medical technology, specifically the seniors.

## Materials and methods

### Study subjects

One hundred and eight senior students completed their internship from August 2018 to July 2021 at the Department of Laboratory Medicine in a teaching hospital in northern Taiwan, which was indicated a convenience sample. Senior students were chosen as the target population because they were interning and had a clearer direction for their future work. There was no time limit for responding to the questionnaire. The learning effectiveness was defined as the student’s exam scores. Thus, two individuals were excluded from the study because they did not have complete exam scores. The relationship among their course performance, exam scores and scales of a written questionnaire for a total of 106 participants were investigated and analyzed. Their age ranged from 19 to 25 years old, containing of 38 male (36%) and 68 female (64%).

### Questionnaire development

The questionnaires were distributed in paper form, which consisted of two sections (Table S1). The first section sought to investigate components that affect learning effectiveness of the study individuals by utilizing four scales: (1) The strength of motivation for medical school, SMMS, comprises 18 items (M1 – M18) [13]; (2) Approaches to Learning and Studying Inventory, ALSI, comprises 18 items (LS1 – LS18) [14]; (3) Perceived medical school stress, PMSS, comprises 10 items (P1 – P10) [15]; (4) Maslach burnout inventory-student survey, MBI-SS, comprises 15 items (F1 – F15) [16]. These questionnaires were used to evaluate the motivation, learning approaches, stress and burnout of students, especially in department of medicine, for a long time. Thus, these 4 questionnaires were selected in this study. The second section of the questionnaire includes 12 questions about individual assessment (S1 – S12) and 2 questions about scenario problems (S13_a – S13_f and S14_a – S14_f), which help to understand each study individual’s personality, social network, specialties, and occupational intents. A commonly-used Likert scale coded in ordered categories 1 to 5 was used to assess each individual’s response to the questions. A Category 1 means “Strongly Disagree”; a Category 2 represents “Disagree”; a Category 3 stands for “Neither Agree nor Disagree”; a Category 4 represents “Agree”; and a Category 5 represents “Strongly Agree”. Each question/item of the questionnaire was coded and analyzed as an ordinal explanatory variable. The outcome variable that we used was the average exam score summarized from course attendance, class performance, quizzes, and written exams, with scores ranging from 0 to 100 for each participant. The complete questionnaire was shown in supplementary Table S1. The reliability and validity of SMMS, PMSS, and MBI-SS in Chinese form had been tested [6, 17, 18]. The Chinese version questionnaire used in this study was firstly translated from the original questionnaire by one author and then translated back into English version by the foreign author. Additionally, the validation of the final questionnaire version in this study was based on the consensus of all authors on the content.

### Ethical approval

The students were informed to do this test, and they could decide to participate or not. Patients’ identifier or personal information was not collected as part of the study. Data were collected as anonymous individuals, and study data were transferred and stored at Department of Laboratory Medicine of Chang Gung Hospital. All participants have written informed consent before filling out the questionnaire. The Institutional Review Board of Chang Gung Hospital has reviewed and approved the study.

### Statistical analysis

The univariate analysis of the outcome with each independent variable was performed by using both Spearman correlation analysis and Kruskal-Wallis tests. Pair-wise Spearman correlation coefficients were first calculated to evaluate the relationship between the outcome (the average exam score) and each ordinal explanatory variable (each questionnaire questions). As performing the Kruskal-Wallis tests, since each ordinal explanatory variable comprises five categories, the average exam scores were compared among the students pertaining to those five categories to investigate whether there is any difference in any of the five groups. These two different methods were used to validate each other and see if consistent results were obtained. The variables with significance level *p* value < 0.05 from the Spearman correlation analysis and Kruskal-Wallis tests of the univariate analysis were identified, and the variables with *p* value < 0.05 in either one of the analyses were used to carry out the multivariate analysis to evaluate the combined contributions of multiple explanatory variables to the outcome. Finally, a multiple linear regression model was built to assess how those explanatory variables affected learning effectiveness altogether. Moreover, the false discovery rate (FDR) *Q* values were calculated to evaluate the proportion of significant tests that will result in false positives [19].

## Results

From the results of the pair-wise Spearman correlation analysis (Table 1), we found that weakened motivation due to uncertainty (M4), thinking the internship occupying too much time (P3), seeing trainings of medical lab technicians as sacrifice of personal lives and interests (P5), competition pressures in the department (P6), stressful major courses (P8), feeling burnt out about learning (F1), and feeling stressed when learning in class (F13) were significantly associated with lower average exam scores (*P* value < 0.05). On the other hand, self-disciplined in learning and studying (LS2), extracurricular studies (LS9), confidence in problem-solving (F3), self-confidence in taking in the contents of the course (F15), willingness to cooperate (S4), cautiousness (S12), preference in traditional learning - sitting in class (S13_f), and preferences in the career of a business commissioner (S14_f) were positively associated with the outcome (*p* value < 0.05). Furthermore, the Q value ranged from 0.170 to 0.374, which meant that about 17.0 to 37.4% of the significant tests will result in false positives (Table 1).

**Table 1:** Variables of significant correlations with the average exam score (*N* = 106)

Variables	Spearman Correlation Coefficient	***P*** value	FDR ***Q*** value
Weakened motivation due to uncertainty (M4)	−0.310	0.001	0.170
Self-disciplined in learning and studying (LS2)	0.244	0.012	0.289
Extracurricular studies (LS9)	0.225	0.021	0.312
Thinking the internship occupying too much time (P3)	−0.200	0.040	0.374
Seeing trainings of medical lab technicians as sacrifice of personal lives and interests (P5)	−0.273	0.005	0.289
Competition pressures in the department (P6)	−0.222	0.022	0.312
Stressful major courses (P8)	−0.213	0.029	0.329
Feeling burnt out about learning (F1)	−0.248	0.011	0.289
Confidence in problem-solving (F3)	0.246	0.011	0.289
Feeling stressed when learning in class (F13)	−0.216	0.026	0.316
Self-confidence in taking in the contents of the course (F15)	0.261	0.007	0.289
Willingness to cooperate (S4)	0.259	0.007	0.289
Cautiousness (S12)	0.200	0.04	0.374
Preference in traditional learning - sitting in class (S13_f)	0.196	0.044	0.374
Preference in the career of a business commissioner (S14_f)	0.217	0.025	0.316

Somewhat similarly, the results of Kruskal-Wallis tests indicated that weakened motivation due to uncertainty (M4, *χ*^*2*^ = 12.055, *df* = 4, *p* value = 0.017, FDR *Q* = 0.289), planning ahead and making good use of time (LS11, *χ*^*2*^ = 9.915, *df* = 4, *P* value = 0.042, FDR *Q* = 0.374), seeing trainings of medical lab technicians as sacrifice of personal lives and interests (P5, *χ*^*2*^ = 12.192, *df* = 4, *P* value = 0.016, FDR *Q* = 0.289), competition pressures in the department (P6, *χ*^*2*^ = 9.939, *df* = 4, *p* value = 0.041, FDR *Q* = 0.374), and preferences in the career of a business director of a biotechnology firm (S14_d, *χ*^*2*^ = 10.132, *df* = 3, *p* value = 0.017, FDR *Q* = 0.289) were associated with the outcome, the average exam score (Table 2). The complete analysis results are presented in supplementary Table S1.

**Table 2:** Significant variables in the Kruskal-Wallis Tests (N = 106)

Variables	Kruskal-Wallis Test Chi square	***P*** value	FDR ***Q*** value
Weakened motivation due to uncertainty (M4)	12.055	0.017	0.170
Planning ahead and making good use of time (LS11)	9.915	0.042	0.289
Seeing trainings of medical lab technicians as sacrifice of personal lives and interests (P5)	12.192	0.016	0.289
Competition pressures in the department (P6)	9.939	0.041	0.312
Preference in the career of a business director of a biotechnology firm (S14_d)	10.132	0.017	0.316

**Fig. 1:**
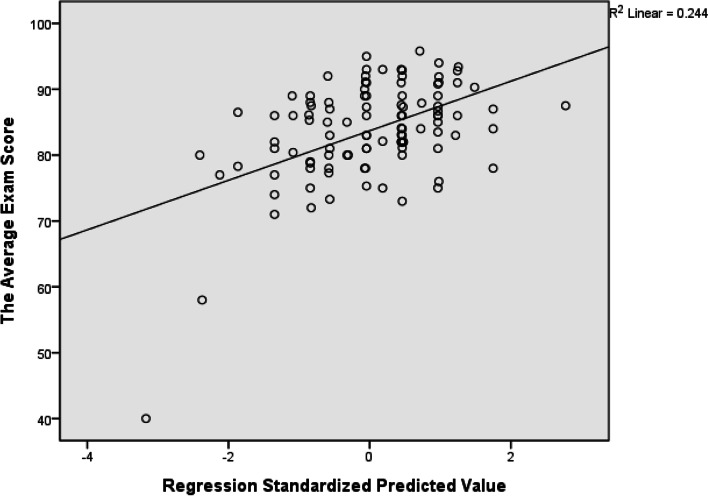
Scatterplot and fitted line in the multiple regression analysis. The linear multiple regression model is of the form: *The Average Exam Score* = 77.793 -0.3*M4 + 0.256*S4 + 0.216*LS9

In the multivariate analysis, a total of 17 variables with *P* value < 0.05 in either of the Spearman correlation analysis or the Kruskal-Wallis test mentioned above, with M4, P5, and P6 showing significance in both, were included in the multiple regression model. A linear model comprised of “weakened motivation due to uncertainty” (M4 standardized *Beta* = − 0.300, *p* value = 0.001), “willingness to cooperate” (S4, standardized *Beta* = 0.256, *p* value = 0.004), and “extracurricular studies” (LS9, standardized *Beta* = 0.216, *p* value = 0.016), were identified (*R* squared = 0.244, *F* test *p* value = 3 × 10^− 6^) as presented in Fig. 1. We found that the model in the form of “***The Average Exam Score*** = 77.793 -0.3*M4 + 0.256*S4 + 0.216*LS9” best described the relationship between multiple explanatory variables and the outcome in our data. That is, the students’ learning effectiveness were mainly affected by whether they had weakened motivation due to uncertainty, whether they were willing to cooperate with others, and whether they would do extracurricular studies after class.

## Discussion

This study sought to investigate factors that might affect learning effectiveness using four scales which represented the strength of students’ motivation for the Department of Medical Technology their approaches to learning and studying, their perceived stress in the department, and MBI-SS in addition to factors indicating the personality, social network, specialties, and occupational intents of the senior students. Our results revealed that“extracurricular studies” and “willingness to cooperate” of the senior students were most crucial to improving their course performance; while “weakened motivation due to uncertainty” impacted negatively on student’s performance.

Supporting the professor’s class content by looking up the evidence (extracurricular studies) and cooperating with others to complete tasks (willingness to cooperate) were positively related to learning effectiveness. It is one of a deep approach that students look for the evidence and try to draw their own conclusion about what they are studying. Deep approach focuses on the meaning of what you are learning, in which the students can link the content they learn from general knowledge, daily experience, and knowledge from other fields or courses [20]. Therefore, the students have a better learning effectiveness if they can integrate information to understand content being taught by seeking meaning, relating ideas, using evidence, and having an interest in ideas rather than rote learning [21] In addition, cooperative learning was beneficial to improve learning effectiveness, which was demonstrated in many studies, no matter in learning genetics, self-efficacy, and conceptions of learning biology [22], Malay language [23], Mathematics [24], or English language [25]. Therefore, cooperating with others can make problem solving easier for students and improve their learning effectiveness. Additionally, our results indicated that “weakened motivation due to uncertainty” was negatively correlated with academic performance. Autonomous motivation is the motivation that is derived from true interest or personal recognition [26] thus students must be motivated, especially students who need professional training for a specific career [27]. Medical technology students’ need for professional training makes motivation a very important factor. However, our students believe that it takes a lot of time to become a medical technologist and the proportion of people who pass the national examination is very low. Consequently, these uncertainties may weaken their motivation to learn.

According to the results, it was known that the medical technology students had better learning results by using deep approach. This may be because the medical technology department is biased towards practical operation during the internship, so it was suggested that educators can manipulate the learning environment, such as through course design, to encourage students to adopt deep learning instead of adopt rote learning [5, 28]. In addition, educators can also divide students into groups and combine deep approach in the classroom. Such interaction not only helps students apply knowledge to learn in an environment that is more similar to the one they will encounter in their future work and life to improve learning effectiveness, but also enables students to become the most active participants in the classroom [29]. However, the uncertainty about the future reduced the learning effectiveness of students, which may be because these students just met the COVID-19 pandemic, and the workload of medical technicians has increased dramatically. Therefore, there were some negative psychological impacts on medical technology students, causing they to doubt whether they will continue to invest in medical technology work in the future. Consequently, we suggested that educators can deeply understand students’ intrinsic motivation, and take corrective measures to increase extrinsic motivation when necessary, such as trying to enlighten them in order to improve their motivation, because both intrinsic and extrinsic motivation affect learning results together [30]. However, this was just our speculation. Whether students really lower their expectations for the future due to COVID-19 still needs further discussion.

The limitation in this study was the reliability of the questionnaire was not tested after translating to Chinese version, we only took the previous studies as reference [6, 17, 18]. Additionally, we did not find out the study suppling an initial evidence for the reliability of Chinese version ALSI. Moreover, the participants were convenience samples, which caused a guaranteed result due to the influence of accidental factors [31]. However, the questionnaires we used were based on the scale published previously, and the validation had been tested. Additionally, the sample size was the major limitation of this study. Although we have made multiple comparison adjustments using FDR Q values to control for the proportion of false positive findings, the overall Q values were above 17%.

## Conclusion

The extracurricular studies and cooperation were significantly improved learning effectiveness; while the uncertainty about future careers would weaken the student’s motivation and worsen their learning effectiveness. Thus, we suggested that the educators can understand the uncertainty of students about the future and assist they in a timely manner. Additionally, the projects that require joint cooperation and discussion need to be given. The most important thing is that students should be able to integrate the learning content instead of rote.

In summary, “extracurricular studies” and “willingness to cooperate” would improving their course grades, while “diminished motivation due to uncertainty” was contrary. Therefore, we suggested that educators can improve the learning effectiveness of medical technology students through curriculum design and catch their inner thoughts.

## Supplementary Information

**Additional file 1.**

## Data Availability

The datasets generated and analyzed during the current study are not publicly available due to the small sample size and possibility of compromising anonymity/individual privacy; however, data may be made available from the corresponding author on reasonable request.
