# Supplementary material for: Exploration of the external and internal factors that affected learning effectiveness for the students: a questionnaire survey
Source: BMC Med Educ. 2023 Jan 23;23:49. doi: 10.1186/s12909-023-04035-4 (PMC9868504; doi:10.1186/s12909-023-04035-4)
Supplement: Supplementary file 1 — Additional file 1. [file 12909_2023_4035_MOESM1_ESM.pdf]

Supplementary Table 1. The complete survey questions and the corresponding analysis results

PART I. Four scales	Coded Variable	Strongly disagree	Disagree	Neutral	Agree	Strongly agree	Spearman Correlation Analysis			Kruskal-Wallis Test			
		1	2	3	4	5	Coefficient	*P* value	FDR *Q* value	Chi square	*P* value	df	FDR *Q* value
**A. The strength of motivation for medical school, SMMS**													
1. I will regret it if I did not study in the medical technology department.	M1						-0.166	0.089	0.4048	3.138	0.535	4	0.7545
2. If I am 95% sure that I won't become the ideal medical lab technician, I will give up studying.	M2						0.051	0.604	0.7856	0.983	0.912	4	0.9734
3. Even if I need to study abroad with an unfamiliar language, I will still choose to study medical biotechnology and laboratory sciences.	M3						-0.068	0.486	0.7511	8.088	0.088	4	0.4048
4. When I found out that it takes a lot of time to become a medical lab technician, I will stop learning.	M4						-0.310	0.001	0.17	12.055	0.017	4	0.289
5. Even if the salary is not high, I will continue to be a medical lab technician.	M5						0.116	0.236	0.5643	3.272	0.513	4	0.7545

6. I have never considered other professions, and just want to be a medical lab technician.	M6	0.043	0.659	0.8238	0.357	0.949	3	0.9778
7. Even if I have to work overtime, I will still choose to become a medical lab technician.	M7	0.141	0.150	0.4554	3.788	0.435	4	0.7043
8. If my test results are not satisfactory, I will give up on the medical technology department and choose to transfer to another department.	M8	0.014	0.884	0.9734	0.474	0.925	3	0.9734
9. If the medical technology department takes a lot of time, I will consider giving up.	M9	-0.103	0.295	0.6338	2.212	0.530	3	0.7545
10. I plan to become a medical lab technician, even if I need to continue my studies in the future.	M10	0.098	0.316	0.6436	6.996	0.136	4	0.4344
11. Even if I do not get accepted into the medical technology department, it will have no effect on me.	M11	-0.170	0.081	0.4048	4.303	0.367	4	0.6621
12. I want to be a medical lab technician, even if I need to consider prioritizing my work over my family.	M12	0.078	0.429	0.7043	6.489	0.166	4	0.4951
13. I will give up on the medical technology department if I find that I am unemployed after graduation.	M13	-0.147	0.132	0.4344	4.143	0.387	4	0.6713

14. If the medical technology department costs a lot of money, I will not study in the medical technology department.	M14	-0.048	0.624	0.7976	2.693	0.610	4	0.7856
15. I must get into the medical technology department even if I need to retake the university entrance exam.	M15	-0.125	0.201	0.5511	1.912	0.591	3	0.7788
16. I originally wanted to become a pharmacist or doctor, but ended up in the medical technology department because of unsatisfactory academic results.	M16	-0.116	0.236	0.5643	2.946	0.567	4	0.7704
17. The reason I am in the medical technology department is to use it as a stepping stone to transfer to another medical department.	M17	-0.078	0.424	0.7043	3.540	0.316	3	0.6436
18. I applied for the medical technology department because of my family's expectations.	M18	-0.106	0.278	0.6138	8.864	0.065	4	0.4048
**B. Approaches to Learning and Studying Inventory, ALSI**								
19. I often forget the material because I am unable to understand the course logically.	LS1	-0.004	0.969	0.981	9.959	0.916	4	0.9734

20. After completing an assignment, I will double-check my reasoning process and whether it is logical.	LS2	0.244	0.012	0.289	6.259	0.100	3	0.4048
21. I am beginning to understand the importance of learning.	LS3	0.059	0.546	0.7608	0.974	0.808	3	0.9219
22. I put a lot of effort into my studies.	LS4	0.154	0.116	0.4344	2.672	0.445	3	0.7118
23. I constantly think about the things that I have learned in my mind.	LS5	-0.008	0.935	0.9734	6.400	0.094	3	0.4048
24. When trying to understand new material, I often connect them to real life examples.	LS6	0.175	0.073	0.4048	4.480	0.214	3	0.5597
25. My learning style is systematic and sequential.	LS7	0.002	0.981	0.981	4.436	0.350	4	0.6538
26. When I read literature, I often come up with lots of ideas, causing me to become deep in thought for long periods of time.	LS8	0.061	0.534	0.7545	0.692	0.875	3	0.9734
27. After class, I will look up information to support the professor’s class content.	LS9	0.225	0.021	0.3117	7.053	0.133	4	0.4344
28. When I exchange ideas with others, I can quickly gather and integrate my own opinions.	LS10	-0.074	0.454	0.7146	4.307	0.230	3	0.5643
29. I plan my study time and make the most of it.	LS11	0.092	0.347	0.6538	9.915	0.042	4	0.374

30. To me, it is important to understand both the general idea as well as the in-depth content when learning things.	LS12	0.159	0.104	0.4112	3.146	0.370	3	0.6621
31. I do not doubt what I have learned.	LS13	-0.094	0.339	0.6538	3.790	0.435	4	0.7043
32. I search for relevant materials outside of the textbook.	LS14	0.186	0.056	0.4048	5.827	0.123	3	0.4344
33. Focusing is not a problem for me, unless I am very tired.	LS15	0.169	0.083	0.4048	7.188	0.126	4	0.4344
34. When I am reading a chapter of course, I will try to find out what the author wants to express.	LS16	0.162	0.097	0.4048	2.924	0.571	4	0.7704
35. If I don’t reach the study goal that I set; I will continue studying.	LS17	0.111	0.256	0.5803	7.431	0.115	4	0.4344
36. If I don’t understand what I have learned, I will try to use a different learning method.	LS18	0.041	0.676	0.8388	3.038	0.386	3	0.6713
**C. Perceived medical school stress, PMSS**								
37. I am worried that I can't stand the duration and responsibility of clinical training.	P1	0.002	0.981	0.981	3.414	0.491	4	0.752
38. I don't know what the professors expect from me.	P2	0.061	0.534	0.7545	4.860	0.302	4	0.6338

39. The internship of the medical technology department occupies all my time and does not leave room for other activities.	P3	-0.200	0.040	0.374	9.413	0.052	4	0.4018
40. I am worried that I cannot learn the relevant knowledge of the medical technology department.	P4	-0.149	0.128	0.4344	5.539	0.236	4	0.5643
41. Medical schools can only train medical lab technicians who can sacrifice their personal lives and interests.	P5	-0.273	0.005	0.289	12.192	0.016	4	0.289
42. The medical technology department is more competitive than I imagined.	P6	-0.222	0.022	0.3117	9.939	0.041	4	0.374
43. I will have a poor learning attitude due to the difficulty of the course.	P7	-0.129	0.189	0.5355	2.803	0.591	4	0.7788
44. The medical technology department is stressful rather than challenging for me.	P8	-0.213	0.029	0.3287	5.120	0.275	4	0.6138
45. Personal finance is one of my sources of stress.	P9	0.008	0.936	0.9734	1.675	0.795	4	0.9132
46. Accommodation is one of the sources of stress for me.	P10	0.009	0.927	0.9734	1.862	0.761	4	0.8861
**D. Maslach burnout inventory-student survey, MBI-SS**								

47. The Department of Medical Technology makes me feel burnt out about learning	F1	-0.248	0.011	0.289	8.216	0.084	4	0.4048
48. Ever since I was in the Department of Medical Technology, my interest in learning has declined.	F2	-0.133	0.173	0.5071	1.926	0.749	4	0.8781
49. I can effectively solve my academic problems.	F3	0.246	0.011	0.289	7.902	0.095	4	0.4048
50. After a full day of class, I feel exhausted.	F4	-0.081	0.407	0.6989	2.923	0.571	4	0.7704
51. My enthusiasm for studying medical technology courses has begun to disappear.	F5	-0.150	0.125	0.4344	2.703	0.609	4	0.7856
52. I believe that my attendance is helpful to the medical technology department.	F6	0.174	0.074	0.4048	4.465	0.347	4	0.6538
53. I am becoming tired of learning.	F7	-0.123	0.21	0.5578	3.277	0.513	4	0.7545
54. I feel that I am an outstanding student.	F8	0.096	0.326	0.652	4.565	0.335	4	0.6538
55. In the course of study, I feel that I have learned a lot of interesting things.	F9	0.08	0.416	0.7043	2.212	0.697	4	0.8524
56. When I wake up in the morning and think that I have to go through a full day of class, I feel tired and I will want to skip classes.	F10	-0.131	0.183	0.5273	4.714	0.318	4	0.6436

57. I am starting to feel negative about going to class.	F11						-0.171	0.079	0.4048	8.674	0.070	4	0.4048
58. I am excited when I reach my learning goals.	F12						0.145	0.137	0.4344	2.564	0.464	3	0.7237
59. For me, class learning has become a source of stress.	F13						-0.216	0.026	0.3157	8.492	0.075	4	0.4048
60. I am not sure about my decision to choose the medical technology department.	F14						-0.164	0.094	0.4048	5.356	0.253	4	0.5803
61. I have confidence in taking in the contents of the course.	F15						0.261	0.007	0.289	6.964	0.138	4	0.4344

**PART II a. Personality assessment**	**Coded Variable**	**Strongly disagree**	**Disagree**	**Neutral**	**Agree**	**Strongly agree**	**Spearman Correlation Analysis**			**Kruskal-Wallis Test**			
		**1**	**2**	**3**	**4**	**5**	**Coefficient**	***P* value**	**FDR *Q* value**	**Chi square**	***P* value**	**df**	**FDR *Q* value**
1. When encountering new tasks, you may be able to increase your skills. Regardless of the outcome, as long as there is a chance to make things better, I will not miss the opportunity to give it a try.	S1						0.035	0.719	0.8669	0.886	0.642	2	0.8145
2. When the amount of work is piling up, I will complete the tasks step by step in an orderly manner.	S2						0.061	0.533	0.7545	1.253	0.740	3	0.8781

3. If something is not done yet, I will stick to my plan, and I will not be anxious.	S3	-0.061	0.537	0.7545	1.929	0.749	4	0.8781
4. When I am assigned a new assignment, I will take the initiative to ask others to work together, because I feel that working together motivates me.	S4	0.259	0.007	0.289	9.413	0.052	4	0.4018
5. When I come across a puzzle problem, my unconscious reaction is to try to work out the solution, only to give up and look at the answers after a long time.	S5	0.045	0.649	0.8173	2.968	0.563	4	0.7704
6. Upon arriving at a social event with strangers, I can naturally and actively mingle with everyone.	S6	0.010	0.919	0.9734	0.843	0.933	4	0.9734
7. When I encounter unreasonable things, I will actively try to change, persuade, and/or change it.	S7	-0.008	0.939	0.9734	0.943	0.918	4	0.9734
8. I can do things at my own pace and finish on time.	S8	0.066	0.500	0.7545	4.351	0.361	4	0.6621
9. Applying creativity to life is easy for me.	S9	-0.012	0.902	0.9734	5.433	0.246	4	0.5729
10. When listening to others, I can pick out what they are trying to say even if they don’t vocally express it.	S10	0.079	0.419	0.7043	2.097	0.718	4	0.8669

11. I hope that I can plan my life as I grow older and gradually complete my dreams without regrets.	S11						0.165	0.091	0.4048	4.404	0.221	4	0.5607
12. Everyone thinks that I am very cautious.	S12						0.200	0.04	0.374	8.077	0.089	4	0.4048

**PART IIb. Scenario problems**	**Coded Variable**	**Most favorite**	**Favorite**	**Neutral**	**Less favorite**	**Least favorite**	**Spearman Correlation Analysis**			**Kruskal-Wallis Test**			
		**1**	**2**	**3**	**4**	**5**	**Coefficient**	***P* value**	**FDR *Q* value**	**Chi square**	***P* value**	**df**	**FDR *Q* value**
13. In response to the adjusted holiday, the professor announced that he/she would need to have a make-up class tomorrow. Which classes would you vote for? Pleas arrange them according to your preference. (1st being most favorite, 6th being least favorite)													
(a) Experiment/laboratory course	S13_a						0.026	0.787	0.9101	0.507	0.973	4	0.981
(b) Case discussion analysis	S13_b						-0.143	0.143	0.442	3.709	0.295	3	0.6338
(c) Innovative career course	S13_c						0.126	0.200	0.5511	4.273	0.370	4	0.6621
(d) Clinical care for patients	S13_d						-0.086	0.380	0.6713	1.512	0.824	4	0.9339
(e) Seminar presentation	S13_e						0.053	0.589	0.7788	7.788	0.100	4	0.4048
(f) Classroom teachings	S13_f						0.196	0.044	0.374	5.925	0.205	4	0.5532

14. The Department of Medical Technology recently invited previous seniors to the school to share their thoughts and experiences in the workplace. At present, there are six professional seniors, please sort them according to their careers or preferences								
(a) Research assistant	S14_a	0.102	0.300	0.6338	0.957	0.916	4	0.9734
(b) Doctor	S14_b	-0.116	0.239	0.5643	3.698	0.448	4	0.7118
(c) A writer who publishes books related to biotechnology	S14_c	0.004	0.967	0.981	2.295	0.682	4	0.8401
(d)Business director of a biotechnology firm	S14_d	0.121	0.221	0.5607	10.132	0.017	3	0.289
(e)Medical lab examiner	S14_e	0.066	0.503	0.7545	1.941	0.747	4	0.8781
(f) Business commissioner	S14_f	0.217	0.025	0.3157	4.523	0.340	4	0.6538
